# Cases of Poisoning

**Published:** 1892-12

**Authors:** J. Hobart Egbert


### CASES OF POISONING.

Hardly any emergency is more common than accidental or intentional poisoning, and scarcely any event or incidental combination of circumstances calls for more immediate action and special treatment. Poisons are substances (material factors) which when introduced into a living organism, are capable, *per se*, of exerting a deleterious, morbid, or deadly action upon that organism. By this definition it will be seen that when speaking of poisons we mean not only drugs which produce toxic effects when taken into the stomach, but also those poisonous agents which act by absorption even when applied to thin and delicate membranes upon the external surface of the body—as septic materials, syphilitic and diphtheritic poisons, etc. ;—also those agents which, when introduced through the respiratory tract, occasion toxic results, and those which must be introduced directly into the circulation through a wound in the flesh or other abrasion—as the poison of insects, the venom of scorpions and serpents, the Indian arrow poison, and that of rabid animals. Space will not permit of more than a brief outline of the best course to pursue in the most common cases of poisoning. The treatment of poisonous wounds, asphyxiation, etc., has already been considered in previous chapters of this series.

In a given case of poisoning, two important questions to consider are : "What was the toxic principle ?"—*i. e.*, What kind of poison is to be dealt with ?—and "How much of the poisonous principle has entered the system ?" These questions cannot always be answered, either by the patient or the companions of the patient. Sometimes the nature of the poison is unknown to the parties concerned ; sometimes the patient who alone knows the desired facts, is unconscious and unable to give information ; again, the strength of a drug administered or the amount of the poisonous agents consumed are often indeterminable ;—and for these and similar reasons it is important to formulate certain general directions universally applicable to cases of poisoning.

First of all induce vomiting or wash out the stomach—providing, of course, the poison has been swallowed and has entered the stomach.

As the stomach-pump and lavage-tube are only to be employed by professional hands, directions for their use are here unnecessary. Emetics—agents which induce vomiting—are both numerous and useful, and play an important role in the treatment of poisoning. The following are available emetics : Common salt (a tablespoonful to half a pint of tepid water), not a very certain emetic, but has the advantage of being always at hand ; ground mustard (tablespoonful to a tumblerful of tepid water) is very good and usually readily procured ; powdered ipecac (thirty grains or more in water) is a good emetic ; sulphate of zinc (twenty grains in water) is a reliable emetic, prompt and quite safe ; tartar emetic (two or three grains in water) is available, but it is slow in action, and usually causes considerable nausea and depression. In cases of poisoning it is not so much a question as to which is the best emetic, as to which can be obtained most speedily. Many people vomit very easily—almost at will—and with them a draught of tepid water—the addition of a few drops of castor oil will enhance its value as an emetic—with the introduction of a finger into the throat, will rapidly produce the desired result. For domestic use either mustard, or ipecac, or both will ordinarily be found the best available emetics for use in cases of poisoning. Cathartics, or purgatives, are to be administered in lieu of emetics when the poison is supposed to be in the intestinal tract.

*Second.*—When the stomach has been duly emptied of its poisonous contents and the poison is known, the next step in the treatment is to administer the proper chemical antagonist and physiological antidote. The proper antidotes for various poisonous agents will be subsequently considered. Numerous atttempts have been made to formulate a standard multiple antidote, that is, to obtain a mixture that will neutralize the toxic action of most, or even all, active poisons. These mixtures are of special value when the nature of the acting poison is unknown. The following is probably the best formula for such a preparation yet offered : Saturated solution of sulphate of iron, 100 parts; water, 800 parts; calcined magnesia, 80 parts ; purified animal charcoal, 40 parts. The iron solution must be kept separately, and the magnesia and animal charcoal should be mixed in another bottle with the water. When required for use, both solutions are poured into one bottle and the whole shaken well together. It is then administered *ad libitum*, a wineglassful at a time. This is a most excellent antidote for preparations of arsenic, zinc and digitalis, rendering them absolutely inert; it delays and partly neutralizes the action of morphia and strychnia, and to a lesser extent deters the action of compounds of mercury. It has no virtue in counteracting the effects of cyanide of mercury, prussic acid, phosphorus, antimony, or the caustic alkalies.

*Third.*—Whenever respiration is yielding to the action of poison, or where it is entirely suspended, it must be continued and sustained by one of the methods of artificial respiration (already considered).

*Fourth.*—The vitality and animal heat are to be maintained by administering stimulants and dilutents, and by electricity and frictions to the surface of the body.

*Fifth.*—The diet is to be restricted to light, nutritious and easily digested food for a few days after recovery.

It is no easy matter to say positively what is the fatal dose of any particular poison. Much depends on the age of the patient, the amount of food in the stomach, the occurrence of copious and early vomiting, the administration of appropriate remedies, etc. It is comparatively easy to ascertain the amounts of given poisons that have produced fatal results; but even here statistics vary, for, aside from the important factors for difference just mentioned, in many of the recorded cases the exact quantity taken is not known, while in others the strength of the preparation is not given.

We now give an abridged classified list of proper antidotes for the various active poisons which most commonly find their way into the system and occasion toxic effects. Unfortunately, we possess but few available chemical antagonists, and hence must rely mainly upon physiological antidotes and general constitutional treatment in relieving most cases of poisoning.

ACIDS.—Acetic, muriatic, nitric (*aqua fortis*), sulphuric (oil of vitriol): Large draughts of soap-suds administered at once; lime-water, chalk and water; magnesia and the carbonates of magnesia; milk, oil, and thick gruel. Carbolic acid: Solution of saccharate of lime; raw white of eggs; give a tablespoonful of castor oil or a wineglassful of olive oil; free use of stimulants (hot brandy and water, etc.) and warmth to extremities. Oxalic and tartaric acids: Lime-water, chalk and water, and castor oil; the administration of potash, soda, ammonia, and the alkaline carbonates must be avoided. Prussic acid (hydrocyanic acid and Scheele's acid): Stimulants, inhalation of ammonia, cold water to head; if patient cannot swallow, give hot coffee per rectum or brandy hypodermically; atropine hypodermically. (Artificial respiration must not be forgotten, nor the necessity for administering an emetic as soon as possible. Space will not permit of calling attention to these essential factors when referring to each separate poison.)

Alkalies (caustic potash, caustic soda, lye, ammonia, hartshorn).—Lemon juice, orange juice, vinegar freely diluted with water; the raw whites of two or three eggs, milk, gruels ; olive oil freely.

Aconite.—Stimulants administered freely ; digitalis ; hot towels and hot water bottles to extremities, mustard poultice over heart.

Alcohol.—Hot strong coffee by mouth or rectum, inhalation of ammonia ; alternate hot and cold douche to head ; rouse patient if insensible and make him move about.

Arsenic (emerald green, Scheele's green, Paris green, rat poison, fly poison, Fowler's solution).—The "multiple antidote" already described is a splendid antidote for all forms of arsenic poisoning, and if at hand should be employed; otherwise, give freshly prepared sesquioxide of iron, made by precipitating tincture of iron with carbonate of soda and filtering through a handkerchief ; it should be given in hot water and in large quantities; or dialyzed iron may be given in half-ounce doses repeatedly. If none of these are at hand, give magnesia in unlimited quantities ; also, castor oil or olive oil frequently in large doses ; albumen (raw eggs); stimulants and warmth to extremities if there is prostration. These poisons are gastro-intestinal irritants, and the resulting condition in intestinal tract requires treatment.

Belladonna (deadly nightshade, atropia, daturia, duboisia, hyoscyamus, stramonium).—Pilocarpine is the chief antidote. Give one-half grain hypodermically ; also, stimulants, such as brandy, sal volatile, etc.; an enema of a pint of hot strong coffee is excellent. Mustard to calves of legs, hot water bottles to feet, alternate hot and cold douche to head, electricity.

Chloral.—Twenty drops of tincture of nux vomica by mouth or rectum; maintain temperature by hot blankets, hot-water bottles, hot bricks, frictions, etc.; stimulants, coffee (by mouth or rectum); artificial respiration on slightest sign of failure of breathing.

Copper (blue vitriol, blue-stone, verdigris).—Administer milk and eggs freely; twenty drops of laudanum by the mouth (for an adult); barley-water, arrowroot, or gruel; no vinegar. (A freshly prepared mixture of sulphide of iron, magnesia, and sulphate of sodium is said to act as a perfect antidote for the salts of copper, corrosive sublimate, and cyanide of mercury.)

Iodine.—Starch and water or raw white of eggs, given freely ; morphine or laudanum if necessary to relieve pain.

Lead (sugar of lead, etc.).—Thirty drops of dilute sulphuric acid or aromatic sulphuric acid in water; or half an ounce of sulphate of magnesia (Epsom salts) dissolved in water; milk, raw white of egg, barley-water. Subsequently, small doses of iodide of potassium should be taken daily to eliminate the drug from the system.

Opium (morphia, soothing syrup, syrup of poppies, Munn's elixir, etc.).—[Sometimes necessary to wash out stomach, where the drug has been swallowed, since in morphine poisoning vomiting is induced with difficulty.] Rouse patient and keep him walking about ; inject a pint of strong coffee into the bowel; pour cold water over the head from a height; give fifteen minims of tincture of belladonna by the mouth, or one-sixtieth of a grain of atropia hypodermically ; give one-sixtieth of a grain of strychnia to sustain respiration, the dose to be repeated once at the end of half an hour; in case of failure of breathing, artificial respiration should be kept up for at least two hours.

Phosphorus (lucifer-matches, rat pastes).—Sulphate of copper is antidotal, hence should be used as an emetic in phosphorus poisoning; give three-grain doses dissolved in water every five minutes until vomiting is induced; if, however, vomiting does not occur after three doses, give a tablespoonful of ground mustard in a tumbler of water. Give mucilaginous drinks and a purgative of half an ounce of Epsom salts. Carefully avoid all oils and fats.

Poisonous fungi (poisonous mushrooms).—After evacuating stomach, give twenty drops of tincture of belladonna, or a hypodermic injection of one-sixtieth of a grain of atropia; give castor oil in full doses ; stimulants ; warmth to extremities and poultices to abdomen.

Strychnia, brucia, nux vomica, vermin killers.—After evacuating stomach, give tannic acid, or gallic acid, or animal charcoal *ad libitum*, to be followed by an emetic; give large doses of bromide of potassium, or cloral hydrate, or both. Do not excite patient.—*J. Hobart Egbert, M. D., in Popular Science Monthly.*

